# 4,5-Bis(isopropyl­sulfan­yl)benzene-1,2-dicarbonitrile

**DOI:** 10.1107/S1600536810008755

**Published:** 2010-03-13

**Authors:** Xingcui Wu, Jianzhuang Jiang, Xiaomei Zhang

**Affiliations:** a School of Chemistry & Chemical Technology, Shandong University, Jinan 250100, People’s Republic of China

## Abstract

In the title compound, C_14_H_16_N_2_S_2_, the C atoms of the aromatic ring, the two cyanide groups and the two S atoms of the isopropyl­sulfanyl groups are almost coplanar [maximum deviation from the mean plane = 0.042 (7) Å]. In the crystal, inversion dimers linked by aromatic π–π stacking occur, with a centroid–centroid separation of 3.7543 (8) Å.

## Related literature

For a related structure and background information on phthalocyanines, see: Zhang *et al.* (2009 ▶). For the synthesis, see: Rey *et al.* (1998 ▶).
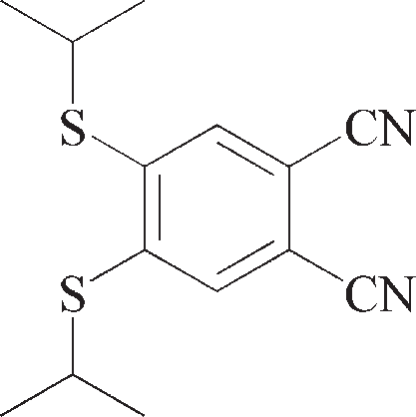


## Experimental

### 

#### Crystal data

C_14_H_16_N_2_S_2_
*M* *_r_* = 276.41
Monoclinic, 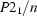

*a* = 10.4929 (7) Å
*b* = 9.3613 (6) Å
*c* = 15.4491 (11) Å
β = 96.467 (1)°
*V* = 1507.87 (18) Å^3^
*Z* = 4
Mo *K*α radiation
μ = 0.34 mm^−1^
*T* = 298 K
0.20 × 0.12 × 0.05 mm

#### Data collection

Bruker APEXII CCD area-detector diffractometer
Absorption correction: multi-scan (*SADABS*; Bruker, 2004 ▶) *T*_min_ = 0.936, *T*_max_ = 0.983
7215 measured reflections
2653 independent reflections
2371 reflections with *I* > 2σ(*I*)
*R*_int_ = 0.016

#### Refinement

*R*[*F* ^2^ > 2σ(*F* ^2^)] = 0.030
*wR*(*F* ^2^) = 0.083
*S* = 1.05
2653 reflections
163 parameters
H-atom parameters constrained
Δρ_max_ = 0.15 e Å^−3^
Δρ_min_ = −0.20 e Å^−3^

### 

Data collection: *APEX2* (Bruker, 2004 ▶); cell refinement: *SAINT* (Bruker, 2001 ▶); data reduction: *SAINT*; program(s) used to solve structure: *SHELXS97* (Sheldrick, 2008 ▶); program(s) used to refine structure: *SHELXL97* (Sheldrick, 2008 ▶); molecular graphics: *SHELXTL-Plus* (Sheldrick, 2008 ▶); software used to prepare material for publication: *SHELXL97*.

## Supplementary Material

Crystal structure: contains datablocks global, I. DOI: 10.1107/S1600536810008755/hb5352sup1.cif

Structure factors: contains datablocks I. DOI: 10.1107/S1600536810008755/hb5352Isup2.hkl

Additional supplementary materials:  crystallographic information; 3D view; checkCIF report

## Figures and Tables

**Fig. 1.**
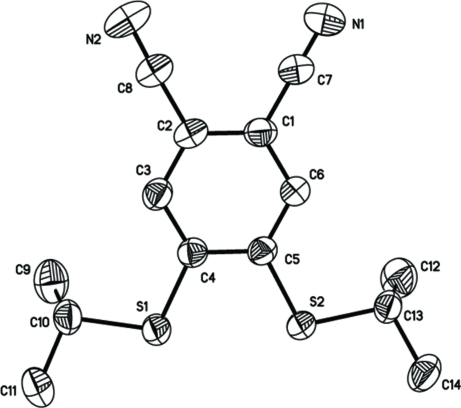
A view of (I) with displacement ellipsoids are drawn at the 30% probability level.

## supplementary crystallographic information

### Comment

As part of our ongoing studies of phthalocyanines (Zhang *et al.*, 2009), we now report the synthesis and structure of the title compound, (I).

As shown in the Fig. 1, the aromatic carbon atoms, two nitrogen atoms and two carbon atoms of two cyanide groups, and two sulfur atoms in the substituted isopropylthio groups build the main skeleton for (I).  The skeleton is almost planar with the maximum deviation from the mean plane of 0.042 (7) Å. The bond distances of cyanide groups are consistent with those in similar compounds  (Zhang *et al.*, 2009).

In the crystal,  inversion dimers (–x, –y, 1–z) linked by aromatic π-π stacking occur, with a centroid-centroid separation of 3.7543 (8)Å.

### Experimental

The title compound was prepared according to the literature (Rey *et al.*, 1998) and colourless plates of (I) were recrystallized from ethanol solution.

### Refinement

All H-atoms bound to carbon were refined using a riding model with distance C—H = 0.93 Å, U_iso_ = 1.2U_eq_ (C) for aromatic atoms, C—H = 0.98 Å, U_iso_ = 1.2U_eq_ (C) for methenyl atoms, and C—H = 0.96 Å, U_iso_ = 1.5U_eq_ (C) for methyl atoms.

### Crystal data

**Table:**

C_14_H_16_N_2_S_2_	*F*(000) = 584
*M**_r_* = 276.41	*D*_x_ = 1.218 Mg m^−^^3^
Monoclinic, *P*2_1_/*n*	Mo *K*α radiation, λ = 0.71073 Å
Hall symbol: -P 2yn	Cell parameters from 4590 reflections
*a* = 10.4929 (7) Å	θ = 2.5–27.4°
*b* = 9.3613 (6) Å	µ = 0.34 mm^−^^1^
*c* = 15.4491 (11) Å	*T* = 298 K
β = 96.467 (1)°	Plate, colorless
*V* = 1507.87 (18) Å^3^	0.20 × 0.12 × 0.05 mm
*Z* = 4	

### Data collection

**Table:**

Bruker APEXII CCD area-detector diffractometer	2653 independent reflections
Radiation source: fine-focus sealed tube	2371 reflections with *I* > 2σ(*I*)
graphite	*R*_int_ = 0.016
Detector resolution: 0 pixels mm^-1^	θ_max_ = 25.0°, θ_min_ = 2.2°
ω scans	*h* = −12→9
Absorption correction: multi-scan (*SADABS*; Bruker, 2004)	*k* = −11→11
*T*_min_ = 0.936, *T*_max_ = 0.983	*l* = −17→18
7215 measured reflections	

### Refinement

**Table:**

Refinement on *F*^2^	Primary atom site location: structure-invariant direct methods
Least-squares matrix: full	Secondary atom site location: difference Fourier map
*R*[*F*^2^ > 2σ(*F*^2^)] = 0.030	Hydrogen site location: inferred from neighbouring sites
*wR*(*F*^2^) = 0.083	H-atom parameters constrained
*S* = 1.05	*w* = 1/[σ^2^(*F*_o_^2^) + (0.0476*P*)^2^ + 0.2613*P*] where *P* = (*F*_o_^2^ + 2*F*_c_^2^)/3
2653 reflections	(Δ/σ)_max_ = 0.001
163 parameters	Δρ_max_ = 0.15 e Å^−^^3^
0 restraints	Δρ_min_ = −0.20 e Å^−^^3^

### Special details

**Table:**

Geometry. All esds (except the esd in the dihedral angle between two l.s. planes) are estimated using the full covariance matrix. The cell esds are taken into account individually in the estimation of esds in distances, angles and torsion angles; correlations between esds in cell parameters are only used when they are defined by crystal symmetry. An approximate (isotropic) treatment of cell esds is used for estimating esds involving l.s. planes.
Refinement. Refinement of *F*^2^ against ALL reflections. The weighted *R*-factor wR and goodness of fit *S* are based on *F*^2^, conventional *R*-factors *R* are based on *F*, with *F* set to zero for negative *F*^2^. The threshold expression of *F*^2^ > σ(*F*^2^) is used only for calculating *R*-factors(gt) etc. and is not relevant to the choice of reflections for refinement. *R*-factors based on *F*^2^ are statistically about twice as large as those based on *F*, and *R*- factors based on ALL data will be even larger.

### Fractional atomic coordinates and isotropic or equivalent isotropic displacement parameters (Å^2^)

**Table:**

	*x*	*y*	*z*	*U*_iso_*/*U*_eq_	
S1	0.19579 (3)	−0.17837 (4)	0.39373 (3)	0.05107 (14)	
S2	0.31036 (3)	0.05041 (4)	0.51139 (2)	0.04640 (14)	
C1	−0.02406 (13)	0.23666 (15)	0.41156 (9)	0.0420 (3)	
C4	0.10593 (12)	−0.02113 (15)	0.39674 (9)	0.0374 (3)	
C5	0.16133 (12)	0.08841 (14)	0.45253 (8)	0.0362 (3)	
C6	0.09506 (13)	0.21542 (15)	0.45910 (9)	0.0418 (3)	
H6	0.1307	0.2873	0.4957	0.050*	
C3	−0.01420 (13)	0.00042 (16)	0.35047 (9)	0.0416 (3)	
H3	−0.0515	−0.0718	0.3148	0.050*	
C2	−0.07884 (13)	0.12884 (16)	0.35707 (9)	0.0408 (3)	
C13	0.35421 (14)	0.20982 (16)	0.57640 (9)	0.0448 (3)	
H13	0.2780	0.2470	0.6001	0.054*	
C7	−0.08952 (15)	0.37074 (18)	0.41839 (11)	0.0538 (4)	
C14	0.45028 (15)	0.1574 (2)	0.65105 (10)	0.0583 (4)	
H14A	0.4111	0.0850	0.6832	0.088*	
H14B	0.4762	0.2360	0.6890	0.088*	
H14C	0.5240	0.1184	0.6280	0.088*	
N1	−0.14093 (17)	0.47715 (17)	0.42345 (12)	0.0778 (5)	
C10	0.10888 (15)	−0.29469 (16)	0.31245 (10)	0.0471 (4)	
H10	0.0191	−0.3013	0.3240	0.057*	
C8	−0.20085 (14)	0.15434 (18)	0.30676 (10)	0.0507 (4)	
N2	−0.29515 (14)	0.1806 (2)	0.26646 (11)	0.0752 (5)	
C12	0.4102 (2)	0.3253 (2)	0.52361 (13)	0.0714 (5)	
H12A	0.3471	0.3552	0.4773	0.107*	
H12B	0.4840	0.2887	0.4995	0.107*	
H12C	0.4349	0.4054	0.5605	0.107*	
C11	0.1728 (2)	−0.43966 (18)	0.32843 (14)	0.0722 (5)	
H11A	0.1680	−0.4681	0.3877	0.108*	
H11B	0.2610	−0.4333	0.3179	0.108*	
H11C	0.1296	−0.5090	0.2898	0.108*	
C9	0.1141 (2)	−0.2436 (2)	0.22038 (12)	0.0700 (5)	
H9A	0.0734	−0.1519	0.2130	0.105*	
H9B	0.0702	−0.3106	0.1805	0.105*	
H9C	0.2019	−0.2358	0.2091	0.105*	

### Atomic displacement parameters (Å^2^)

**Table:**

	*U*^11^	*U*^22^	*U*^33^	*U*^12^	*U*^13^	*U*^23^
S1	0.0376 (2)	0.0453 (2)	0.0673 (3)	0.00601 (15)	−0.00753 (17)	−0.01699 (17)
S2	0.0365 (2)	0.0456 (2)	0.0536 (2)	0.00658 (15)	−0.01006 (16)	−0.00971 (16)
C1	0.0399 (7)	0.0423 (8)	0.0430 (7)	0.0060 (6)	0.0014 (6)	0.0032 (6)
C4	0.0323 (7)	0.0402 (7)	0.0398 (7)	−0.0001 (5)	0.0044 (5)	−0.0015 (6)
C5	0.0315 (7)	0.0403 (7)	0.0363 (7)	0.0011 (5)	0.0020 (5)	−0.0001 (5)
C6	0.0409 (8)	0.0401 (8)	0.0426 (8)	0.0028 (6)	−0.0031 (6)	−0.0029 (6)
C3	0.0354 (7)	0.0453 (8)	0.0433 (7)	−0.0034 (6)	0.0005 (6)	−0.0035 (6)
C2	0.0320 (7)	0.0489 (8)	0.0406 (7)	0.0008 (6)	0.0005 (6)	0.0062 (6)
C13	0.0383 (7)	0.0513 (9)	0.0434 (8)	0.0002 (6)	−0.0017 (6)	−0.0117 (6)
C7	0.0507 (9)	0.0505 (9)	0.0569 (9)	0.0110 (7)	−0.0083 (7)	0.0004 (7)
C14	0.0425 (8)	0.0797 (12)	0.0499 (9)	0.0037 (8)	−0.0078 (7)	−0.0122 (8)
N1	0.0790 (11)	0.0594 (10)	0.0895 (12)	0.0271 (9)	−0.0150 (9)	−0.0073 (8)
C10	0.0425 (8)	0.0420 (8)	0.0561 (9)	−0.0071 (6)	0.0025 (6)	−0.0093 (7)
C8	0.0384 (8)	0.0609 (10)	0.0513 (9)	0.0004 (7)	−0.0022 (7)	0.0051 (7)
N2	0.0455 (8)	0.1017 (13)	0.0736 (10)	0.0064 (8)	−0.0150 (7)	0.0103 (9)
C12	0.0734 (13)	0.0612 (11)	0.0777 (13)	−0.0156 (9)	0.0006 (10)	0.0011 (9)
C11	0.0770 (13)	0.0440 (10)	0.0921 (14)	0.0008 (9)	−0.0066 (11)	−0.0183 (9)
C9	0.0839 (13)	0.0701 (12)	0.0574 (10)	−0.0124 (10)	0.0142 (9)	−0.0105 (9)

### Geometric parameters (Å, °)

**Table:**

S1—C4	1.7515 (14)	C7—N1	1.140 (2)
S1—C10	1.8265 (15)	C14—H14A	0.9600
S2—C5	1.7545 (13)	C14—H14B	0.9600
S2—C13	1.8284 (15)	C14—H14C	0.9600
C1—C6	1.3909 (19)	C10—C9	1.507 (2)
C1—C2	1.396 (2)	C10—C11	1.521 (2)
C1—C7	1.440 (2)	C10—H10	0.9800
C4—C3	1.3920 (19)	C8—N2	1.135 (2)
C4—C5	1.4211 (19)	C12—H12A	0.9600
C5—C6	1.3868 (19)	C12—H12B	0.9600
C6—H6	0.9300	C12—H12C	0.9600
C3—C2	1.390 (2)	C11—H11A	0.9600
C3—H3	0.9300	C11—H11B	0.9600
C2—C8	1.441 (2)	C11—H11C	0.9600
C13—C12	1.512 (2)	C9—H9A	0.9600
C13—C14	1.525 (2)	C9—H9B	0.9600
C13—H13	0.9800	C9—H9C	0.9600
			
C4—S1—C10	106.90 (7)	H14A—C14—H14B	109.5
C5—S2—C13	105.88 (7)	C13—C14—H14C	109.5
C6—C1—C2	119.97 (13)	H14A—C14—H14C	109.5
C6—C1—C7	119.57 (13)	H14B—C14—H14C	109.5
C2—C1—C7	120.46 (13)	C9—C10—C11	111.96 (15)
C3—C4—C5	119.46 (13)	C9—C10—S1	113.04 (11)
C3—C4—S1	124.56 (11)	C11—C10—S1	104.09 (11)
C5—C4—S1	115.97 (10)	C9—C10—H10	109.2
C6—C5—C4	119.26 (12)	C11—C10—H10	109.2
C6—C5—S2	124.12 (10)	S1—C10—H10	109.2
C4—C5—S2	116.61 (10)	N2—C8—C2	176.87 (19)
C5—C6—C1	120.80 (13)	C13—C12—H12A	109.5
C5—C6—H6	119.6	C13—C12—H12B	109.5
C1—C6—H6	119.6	H12A—C12—H12B	109.5
C2—C3—C4	120.59 (13)	C13—C12—H12C	109.5
C2—C3—H3	119.7	H12A—C12—H12C	109.5
C4—C3—H3	119.7	H12B—C12—H12C	109.5
C3—C2—C1	119.90 (12)	C10—C11—H11A	109.5
C3—C2—C8	121.00 (14)	C10—C11—H11B	109.5
C1—C2—C8	119.08 (13)	H11A—C11—H11B	109.5
C12—C13—C14	111.97 (14)	C10—C11—H11C	109.5
C12—C13—S2	112.10 (11)	H11A—C11—H11C	109.5
C14—C13—S2	104.90 (11)	H11B—C11—H11C	109.5
C12—C13—H13	109.3	C10—C9—H9A	109.5
C14—C13—H13	109.3	C10—C9—H9B	109.5
S2—C13—H13	109.3	H9A—C9—H9B	109.5
N1—C7—C1	179.6 (2)	C10—C9—H9C	109.5
C13—C14—H14A	109.5	H9A—C9—H9C	109.5
C13—C14—H14B	109.5	H9B—C9—H9C	109.5
